# An Alternative Psychophysical Diagnostic Indicator of the Aging Eye

**DOI:** 10.1155/2019/2036192

**Published:** 2019-11-03

**Authors:** John D. Rodriguez, Garrick Wallstrom, Divya Narayanan, Donna Welch, Mark B. Abelson

**Affiliations:** ^1^Ora, Inc., Andover, MA, USA; ^2^Statistics & Data Corporation, Tempe, AZ, USA; ^3^Harvard Medical School, Department of Ophthalmology, Boston, MA, USA

## Abstract

**Purpose:**

Impaired adaptation to changes in lighting levels as well as mesopic visual function is a common complaint in those over the age of 65. The use of photostress is a well-established method to test the adaption rate and the response of the visual cycle. In this study, we test visual function recovery to mesopic luminance stimuli following a long duration photostress in young and elderly subjects. If successful in strongly differentiating aging macular function, these methods may also be useful in the study of pathologies such as age-related macular degeneration.

**Methods:**

A group of 12 older normal subjects (mean age 75.1 ± 4.79) and a control group of 5 younger normal subjects (mean age 26.2 ± 4.19) were subjected to macular photostress using the OraLux photostress system. The OraLux system provides a diffuse light source bleaching 84% of cone photopigment while maintaining an exposure safety factor of 200 times less than the maximum safe exposure. After each photostressing session, macular recovery was tracked using a foveal, variable contrast, flickering stimulus of mean luminance in the high mesopic range. Recovery was tracked for 300 seconds. The endpoint was time to recovery to each individual's baseline sensitivity as determined by two static sensitivity trials prior to photostress.

**Results:**

Proportional hazards analysis of recovery time yielded a statistically significant difference between the older group and the young group (HR = 0.181; *p*=0.0289). The estimated hazard ratio of 0.181 indicates that older subjects return to baseline at less than one-fifth the rate of younger subjects. The hazards ratio remained statistically significant after adjusting for visual acuity (HR = 0.093; *p*=0.0424).

**Conclusion:**

Photostress recovery of flicker sensitivity under mesopic conditions is a strong differentiator of aging macular function. This agrees with subject-reported complaints in reduced luminance conditions after exposure to bright lights such as night driving. The qualitative similarity between the aging retina and changes in early AMD suggests that flicker recovery following photostress may be useful as a surrogate endpoint in AMD clinical trials.

## 1. Introduction

The use of photostress is a well-established method to test the adaption rate of the visual system and the response of the visual cycle [1, 2]. The regeneration of photopigment can be impaired either due to retinal disease [3–6] or as a normal consequence of aging [7]. In the elderly, difficulties in adaptation to changes in lighting levels as well as mesopic luminance visual function are a common quality of life complaint, particularly regarding night driving [8].

Age-related changes in the eye may arise from several sources and affect visual function. In particular, changes in the cornea and lens have strong effects on visual acuity. Age-related changes in the retina are less well studied. The use of flickering stimuli is less dependent on refractive error or straylight and is useful for this purpose [9]. The addition of photostress with a flicker endpoint is thus a possibly useful stress test for assessing the health of the aging retina.

Retinal diseases may also result in diminished robustness of the visual cycle and adaptation to changing light levels. Both age-related macular degeneration (AMD) and diabetic retinopathy have been shown to be important examples [5, 6, 10–14]. The use of photostress in this context is as a stress test of the visual cycle analogous to the widely used cardiovascular stress test in order to more easily detect pathology in the early disease state.

Both aging and AMD have been shown to result in rod photoreceptor loss and diminished sensitivity in short wavelength (blue) photoreceptors [15, 16]. Thus, in addition to improved understanding of adaptive visual processes in the elderly, the study of the normal aging retina may yield insight into retinal disease as well.

A considerable number of studies using photostress have been reported since the initiation of this method. Studies have included both normal aging subjects [17–26] and subjects with retinal disease [5, 6, 10–14, 22]. A common difficulty of many of these studies has been inconsistency of the hardware apparatus used for the bleaching process [21]. Following the bleach process, recovery has been determined using various outcomes, most commonly recovery of visual acuity.

In recent studies, the introduction of computer-based stimuli using central foveal disks or blobs with sinusoidal time-varying flickering stimuli has permitted much greater flexibility in testing methodology [9, 27, 28]. As a stand-alone endpoint, foveal flicker sensitivity has shown declines with age in both photopic and mesopic luminance levels [9, 29]. In addition, recent studies of AMD subjects have found that computer-based methods using variable contrast flickering stimuli are a particularly useful and effective endpoint [27, 28].

The purpose of the present study is to improve the understanding of photostress recovery using a time-varying flickering stimulus in an aged population. Results are compared to a group of young subjects. Within the context of clinical trials, these results may be useful to provide control data for the clinical application of variable contrast flickering stimuli to pathological conditions such as AMD.

## 2. Methods

### 2.1. Subjects

Two groups of subjects were enrolled: young subjects (early thirties and younger) and older subjects (60 years of age and older). All subjects were recruited from a single general ophthalmology practice. All subjects provided written informed consent, and study protocols were approved by a properly constituted Institutional Review Board (Alpha IRB, San Clemente, CA). The study was conducted in accordance with the ethical principles of the Declaration of Helsinki.

All subjects provided medical and ocular history and were tested for ETDRS visual acuity at baseline and following photostress. Retinal imaging, including OCT and dilated fundus photography, was used to confirm absence of retinal disease. All subjects, young and old, were required to have no evidence or history of ocular disease or any medical condition that the investigator felt put the subject at significant risk, confounded the study results, or interfered significantly with study participation. All subjects were required to present with visual acuity of 20/25 or better in at least one eye (the study eye). If different, the eye with better visual acuity was chosen as the study eye. If both eyes were tested at equal visual acuity, the right eye was chosen as the study eye. Data were collected on all qualified eyes.

### 2.2. Baseline Cone Function and Recovery

Baseline retinal cone photoreceptor sensitivity was measured using a Python-based software program developed in-house for this study. A foveal, flickering sinusoidal time-varying stimulus of approximately 2° visual angle was presented on a background of luminance intensity in the upper mesopic range. The luminance range chosen was based on the earlier work of Collins and Brown in a study of an AMD population [13, 14]. The contrast between maximum stimulus brightness and background was the outcome variable. The flicker frequency of the target stimulus was based on a range chosen to bracket the sensitivity of the human visual system to this stimulus [30]. Based on previous studies of AMD subjects [27, 28], we investigated stimuli at several frequencies. The stimulus was viewed monocularly from a distance of one meter. The visual task was the subject identifying the presence of the stimulus. All subjects were first required to complete a demonstration run to ensure that each subject could properly identify the presence or absence of the stimulus based on ten trials. Subjects were required to correctly identify the presence or absence of the stimulus at least 80% of the time. The sensitivity of the subject to the stimulus was first determined before photostress as a baseline based on two trials. After assessing baseline flicker threshold, photostress was applied as described below. Recovery was measured by the subjects identifying the presence of the stimulus through the resulting afterimage. The test was terminated five minutes after exposure to the bleaching lamp. The study outcome was the time to return to baseline sensitivity. A simple staircase procedure was used to track recovery following photobleach.

During the photostress recovery process, fixation lines were presented to assist the subject in maintaining gaze on the area of the screen in which the stimulus was presented. In addition, a fixation circle of diameter corresponding to the bleaching area was presented. The subject was instructed to center the resulting afterimage within the circle.

A second measurement of ETDRS visual acuity was made following photostress testing as a safety check. In addition, subjects who had not returned to baseline after five minutes were retested one hour following photostress to confirm recovery of visual function before visit termination.

### 2.3. Photobleach Procedure

The photostress procedure was performed using a custom-designed full-spectrum diffused fluorescent light source (Ora LUX) [31]. The level of retinal irradiance of the Ora LUX source yields at least 84% cone photoreceptor bleach. The center of the bleaching light was aligned with the center of each subject's gaze in the vertical and horizontal directions as required. Subjects were instructed to maintain their gaze on the center of the bleaching light and to avoid squinting, but were allowed to blink normally during the procedure. Compliance by the subject was monitored by the technician. A safety analysis found that the maximum exposure level of the Ora LUX light source was 200 times less than maximum permissible exposure (at least 2000 times less actual damage level) based on accepted safety standards for thermal and photochemical mechanisms [32]. For additional safety in this sensitive group, exposure is less than 90 seconds and distance greater than 12 inches.

### 2.4. Statistical Methods

Unadjusted group means were compared using pooled two-sided two-sample *t*-tests if the folded *F*-test for equality of variances was not statistically significant at *α* = 0.05 level; otherwise, the Satterthwaite approximation was used.

The proportion of subjects that failed to return to baseline within 5 minutes were calculated and compared between groups using Fisher's exact test. Failure rates using all qualified eyes were compared using logistic regression with a random subject factor to account for the correlation between eyes. Recovery times were analyzed using proportional hazards regression and tested using a Wald test. Proportional hazards analyses of all qualified eyes included a random subject factor. Kaplan–Meier product limit estimation was used to generate recovery time curves.

Statistical analyses were conducted using SAS 9.4, with PROC FREQ, PROC GENMOD, PROC PHREG, and PROC LIFETEST.

## 3. Results

### 3.1. Demographics and ETDRS BCVA

The mean age for the older group was 75.1 ± 4.79 years (67.0–83.3) (4M, 8F). For the young group, the mean age was 26.2 ± 4.19 years (19.5–30.0) (2M, 3F). At baseline, mean ETDRS best-corrected visual acuity for the younger group was −0.04 ± 0.055 and for the older group 0.17 ± 0.167. The group means were statistically different based on the Satterthwaite *t*-test (*p*=0.0017).

### 3.2. Baseline Flicker Sensitivity

Mean normalized baseline flicker sensitivity (SD) was 0.11 (0.161) for the older group and 0.05 (0.026) for younger. The difference was not statistically significant, *p*=0.2077.

### 3.3. Photostress Recovery

**Figure 1:**
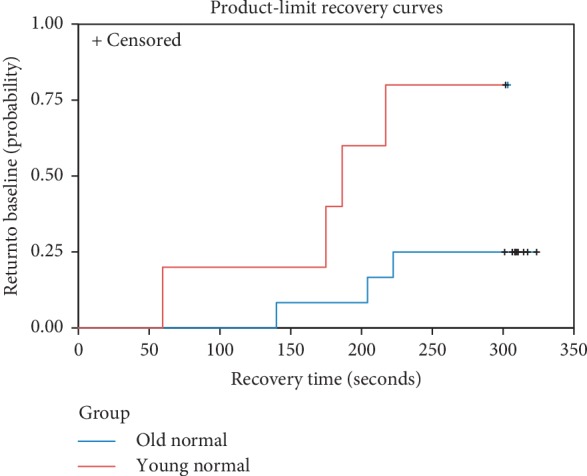
Kaplan–Meier recovery curves for the older group (blue) and the younger group (red).

In the older group, 9 out of 12 (75%) study eyes failed to return to baseline within 5 minutes, compared to 1 out of 5 (20%) in the young group (*p*=0.1007) (Figure 1). In the analysis of failure rates using all qualified eyes, the odds of failing to return to baseline are 1.92 in the older group compared to 0.25 in the young group (OR = 7.69; *p*=0.0203).

The proportional hazards analysis of recovery time yielded a statistically significant difference between the older group and the young group (HR = 0.181; *p*=0.0289). The estimated hazard ratio of 0.181 indicates that older subjects return to baseline at less than one-fifth the rate of younger subjects. Luminance (*p*=0.2093) and frequency (*p*=0.3665) of the stimulus were not statistically significant and were removed from the model. The hazards ratio remained statistically significant after adjusting for visual acuity (HR = 0.093; *p*=0.0424). In the all qualified eyes analysis, the estimated hazard ratio was HR = 0.134 (*p*=0.0046) and HR = 0.032 (*p*=0.0022) after adjusting for visual acuity.

## 4. Discussion

Previous visual psychophysical-based studies of photostress recovery in both normal and AMD subjects have primarily used visual acuity recovery as an endpoint [17–26]. Various approaches have been used to apply photostress to the subject's visual system which has been identified as a significant factor in test variability [21]. The most important differentiator between these approaches has been bleaches using a photoflash stimulus of duration of several milliseconds and long-duration “equilibrium” bleach of several seconds or more [33, 34]. For bleaching cone photoreceptors, an equilibrium bleach has been shown to be preferable in order to deplete local stores of photopigment derived from the retinoid present in the Müller cells [35]. Our approach follows this methodology using a custom-designed light source (Ora Lux) to provide a bleach of at least 84% of cone photoreceptors while maintaining safe levels of exposure [31, 36].

Results of at least eight previous studies of healthy subjects over a broad range of age, based on visual psychophysical outcomes, have been published [19–26]. Six of these studies found a significantly longer mean recovery time in older subjects [19–24]. Reported recovery time of oldest to youngest subjects varied considerably from as little as 15% to over 90%. Since these studies used a recovery endpoint of visual acuity, one must be cautious in a direct comparison to the results presented here.

In early age-related macular degeneration, outer retinal metabolism is known to be compromised [37]. Since the detection of a flickering stimulus imposes a higher metabolic requirement than does a static stimulus, flicker detects functional change at an earlier stage of the disease [38]. The variable contrast flickering stimulus investigated here was selected based on the effectiveness of showing group differences between normal and AMD subject groupings [9, 27, 28]. Based on previous results from the literature [27, 28], we have investigated several target frequencies and luminance levels. These changes are designed to provide optimal differentiation between normal and AMD groups as well as improve reliability. Sensitivity to foveal flickering stimuli has been shown to decrease with age [9]. However, in this study, mean baseline sensitivity between young and old was not found to be statistically different. Nevertheless, the addition of photostress to the baseline stimulus resulted in an increased separation between groups which was found to be significant. Since retinal changes in AMD appear to share some similarities to age-related changes, we would expect that young normal and older normal subjects should show easily detectable differences. The differences found here in this study suggest that this is the case.

## 5. Conclusion

In contrast to previous studies on photostress in normal subjects, in which recovery endpoints based on visual acuity were used, the recovery endpoint used here is a flickering foveal blob presented on a computer screen with mesopic luminance background. Unlike visual acuity, this endpoint is particularly useful for assessing the health of the aging retina due to its relative insensitivity to defects in the ocular media. The combination of photostress and flicker constitute a retinal stress test which also stresses the visual cycle and retinal metabolism. We find that using a mesopic flicker recovery target followed by our high bleach photostress system strongly differentiates aging macular function. Inconsistent and nonstandardization of the bleach process used in previous studies has been found to result in high variability in outcomes [21]. The use of a consistent bleaching process in this study, bleaching a high percentage of cone photoreceptors (84%), should allow for reduced variability and more efficient clinical trials. Moreover, the flicker endpoint described here extends previous work by incorporating reduced luminance background. This is in accord with well-known difficulties reported by both AMD and older normal subjects under mesopic luminance levels. This study shows that a flicker target with high bleach photostress methodology is sensitive to the retina/macular changes that occur as a result of age. Perhaps this same methodology would differentiate between age-related retinal changes and retinal changes due to AMD. In clinical trials, visual function endpoints, such as the variable contrast flickering endpoint may provide a useful supplement to current endpoints in studies of early AMD.

## Data Availability

The data used in this study are contained in the supplementary data file.

## Supplementary Materials

Supplementary Materials The data used in this study are contained in the supplementary data file. The data are organized with one subject eye per row. The columns are as follows: ID: subject ID number; age: subject age in years; age group: young for age <31.0 and older for age ≥60.0; gender: F for female and M for male, SEYE: subject study eye; OD or OS QEYE: subject qualifying eye(s); OD, OS, or OU EYE: subject eye tested; OD or OS lum: luminance of stimulus; 30 or 60 freq: frequency of stimulus; 4, 14, or 30 Hz baseThr: nonnormalized baseline threshold; baseline_Fflicker: normalized baseline threshold; return_time: time to return to baseline; blank for eyes that do not return to baseline within the allotted time; time_last: last stimulus time; ETDRS: ETDRS (logMAR).
